# Cancer Cachexia and MicroRNAs

**DOI:** 10.1155/2015/367561

**Published:** 2015-10-04

**Authors:** Rodolfo Gonzalez Camargo, Henrique Quintas Teixeira Ribeiro, Murilo Vieira Geraldo, Emídio Matos-Neto, Rodrigo Xavier Neves, Luiz Carlos Carnevali, Felipe Fedrizzi Donatto, Paulo S. M. Alcântara, José P. Ottoch, Marília Seelaender

**Affiliations:** ^1^Cancer Metabolism Research Group, Institute of Biomedical Sciences, University of São Paulo, Avenida Professor Lineu Prestes 1524, Cidade Universitária, 05508-000 São Paulo, SP, Brazil; ^2^NAPmiR—miRNA Research Group, University of São Paulo, Avenida Professor Lineu Prestes 1524, Cidade Universitária, 05508-000 São Paulo, SP, Brazil; ^3^Department of Clinical Surgery, University of São Paulo, Avenida Professor Lineu Prestes 2565, Cidade Universitária, 05508-000 São Paulo, SP, Brazil

## Abstract

Cancer cachexia is a paraneoplastic syndrome compromising quality of life and survival, mainly characterized by involuntary weight loss, fatigue, and systemic inflammation. The syndrome is described as a result of tumor-host interactions characterized by an inflammatory response by the host to the presence of the tumor. Indeed, systemic inflammation is considered a pivotal feature in cachexia progression and maintenance. Cytokines are intimately related to chronic systemic inflammation and the mechanisms underlying the release of these factors are not totally elucidated, the etiology of cachexia being still not fully understood. Therefore, the understanding of cachexia-related mechanisms, as well as the establishment of markers for the syndrome, is very relevant. MicroRNAs (miRNAs) are a class of noncoding RNAs interfering with gene regulation. Different miRNA expression profiles are associated with different diseases and inflammatory processes. miRNAs modulate adipose and skeletal muscle tissue metabolism in cancer cachexia and also tumor and tissue derived inflammation. Therefore, we propose a possible role for miRNAs in the modulation of the host inflammatory response during cachexia. Moreover, the establishment of a robust body of evidence in regard to miRNAs and the mechanisms underlying cachexia is mandatory, and shall contribute to the improvement of its diagnosis and treatment.

## 1. Introduction

Cachexia is a wasting syndrome for which descriptions may be found as far as 2000 years ago [1], and is a consequence of cancer and other diseases, such as chronic obstructive lung disease, multiple sclerosis, congestive heart failure, tuberculosis, and AIDS, among others, with a high impact on quality of life [2]. In this review, we focus primarily on cancer cachexia, which affects approximately half of all patients with cancer. In advanced stages, this figure rises up to 80% [3, 4]. The condition compromises the responsiveness to cancer treatment and represents, per se, the direct cause of death of up to 20% of all patients [5].

The syndrome is characterized by unintentional significant reduction in body weight and, among other symptoms, reduced energy intake, fatigue, systemic inflammation, and metabolic abnormalities are frequently reported [6].

**Figure 1:**
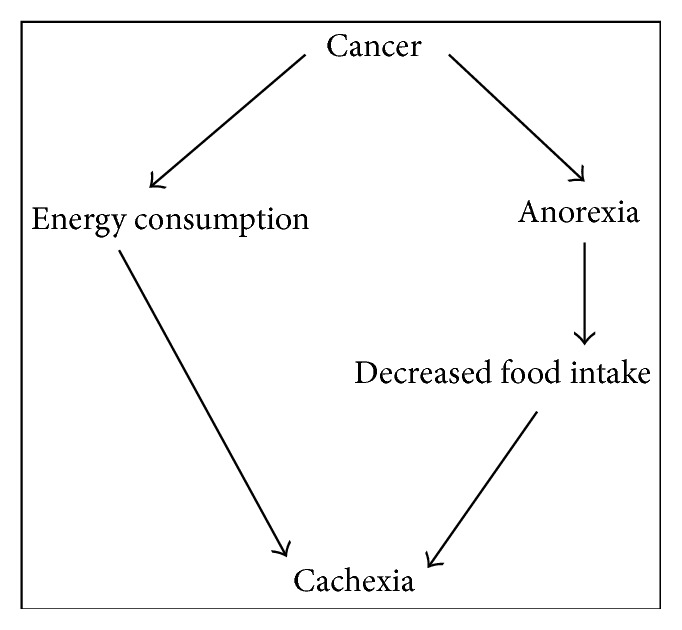
Traditional view of cachexia as discussed by Tisdale [8].

**Figure 2:**
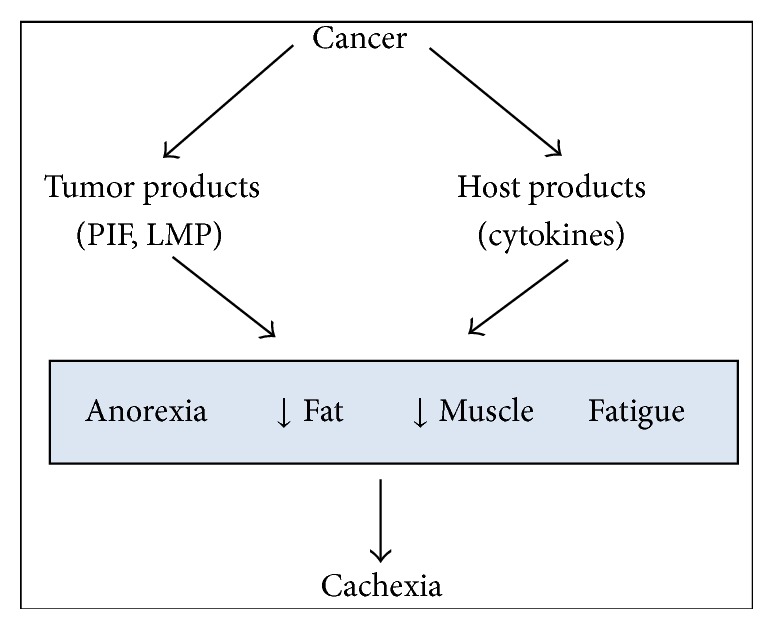
Emerging view of cachexia as proposed by Tisdale [8].

Despite the long search for etiologic factors underlying cachexia, and the fact that many scientific efforts have been devoted to its understanding, researchers agree that “we are still a long way from knowing the whole truth about the exact mechanisms behind its etiology” [7], which makes it very hard to diagnose and treat the syndrome, frustrating physicians and patients. The most widely accepted hypothesis is that cachexia would appear as the result of tumor-host interactions (Figure 1) [8], being deeply related to the increase and release of proinflammatory factors (Figure 2) [8, 9].

## 2. Cachexia Definition and Main Symptoms

Marked weight loss is the central symptom in many of the proposed diagnostic criteria [10–12]. In 2011, a definition consensus for cachexia suggested the existence of different degrees of the syndrome. The syndrome would thus develop through three different and specific stages: precachexia, when anorexia and metabolic changes may be observed before weight loss; cachexia itself, with a weight loss ≥ 5% or BMI ≤ 20 and weight loss ≥ 2% or sarcopenia and weight loss > 2% and often reduced food intake and systemic inflammation; and, finally, refractory cachexia, in which survival expectance usually does not exceed three months [11].

## 3. Cachexia and Peripheral Tissues

In the wasting scenario in cancer cachexia neuroendocrine changes play an important part, provoking early satiety and aversion to food and leading to undernutrition [13]. These, combined with diminished food absorption and hypermetabolism, lead to a negative energy balance [14, 15] and contribute to the loss of mass, specially of adipose tissue and of skeletal muscle [16].

Nevertheless, peripheral tissues are highly affected by cachexia even before the presence of anorexia. Thus, the loss of adipose tissue and skeletal muscle mass precedes any decrease in food intake, which at the initial period of wasting can be normal or even increased [17, 18]. These tissues exhibit impaired homeostasis and altered metabolism, resulting in increased lipolysis in the adipose tissue and augmented proteolysis in the skeletal muscle. The white adipose tissue seems to be importantly adding to the inflammatory status in cachexia. Several studies showed that circulating levels of cytokines are altered in cachectic patients [19–22]. These cytokines elicit an inflammatory response in the adipose tissue, which then releases chemoattractant proteins, which in turn will recruit immune cells from the blood stream; these cells infiltrate the tissue, provoking further release of proinflammatory mediators. As a consequence, lipolysis is activated, causing adipocytes and immune cells to secrete, in a vicious cycle, proinflammatory mediators such as tumor necrosis factor (TNF-alpha), interleukin- (IL-) 1*β*, interferon-gamma (INF-gamma), and IL-6. These cytokines may reach other tissues through the circulation and are associated with increased muscle catabolism and reduced muscle protein synthesis. On the other hand, the high concentration of circulating free fatty acids is sensed by the liver. This organ responds by increasing the uptake of these substrates, which, ultimately, may lead to the onset of steatosis and the induction of acute phase protein secretion. These changes contribute to the systemic onset of the so-called metabolic chaos.

## 4. miRNAs

Recently, changes in metabolism and in aspects of the inflammatory response have been found to be modulated by miRNAs, which are small noncoding RNAs of approximately 19–25 nucleotides (nt), known to be regulatory molecules for some of the most important levels of genome function, including chromatin structure, chromosome segregation, transcription, RNA processing, RNA stability, and translation [23].

These molecules are widely found in organisms including plants, nematodes, fruit flies, and mammals and are highly conserved among evolution [24]. miRNA biogenesis involves the transcription of genomic DNA by RNA polymerase II to produce primary miRNA transcripts (pri-miRNA). In sequence, the Drosha-DGCR8 RNase complex initiates miRNA maturation through the cleavage of a stem loop into the primary transcript. This generates a 60- to 70-nucleotide-long miRNA precursor, the “pre-miRNA,” characterized by the presence of an overhang of 2-3 nucleotides, still in the nucleus [25]. The newly produced pre-miRNA is then transported to the cytoplasm by exportin5 and processed to a double-stranded RNA molecule of about 19 to 25 nucleotides in length by yet another enzyme, the Dicer. Once incorporated into the effector complex miRISC (miRNA-induced silencing complex), one strand of the recently produced RNA molecule remains as a mature miRNA [26, 27], while the other strand may be either degraded, incorporated into another miRISC, or exported to the periphery by exosomes to exert its effects in a paracrine or endocrine way [28, 29]. The complex miRISC, together with the recently incorporated mature miRNA, acts directly on the mRNA to repress the translation of target genes by cleavage (perfect or near-perfect binding) or by forming a “hairpin” in the 3′UTR, through imperfect base pairing [30]. The binding site may also not be in the 3′UTR, but in the ORF or 5′UTR region of the target [31].

According to the miRBase [32], over 6,000 miRNA genes were identified in more than 223 known species, including viruses, plants, fungi, and animals. In humans, the number of miRNAs reaches up to 1500 (access in December 2014). Computational analysis estimates that more than 50% of human protein-coding genes are putatively regulated by miRNAs [33].

## 5. miRNAs, Peripheral Tissues, Cachexia, and Inflammation

The expression of miRNAs is highly dependent on tissue type, metabolic status, and presence of disease. Several studies describe miRNAs as important regulators of biological processes as cellular differentiation, proliferation, tissue development, and cell-type specific function and homeostasis. Nowadays, an increased number of diseases have been found to be associated with altered miRNAs expression [34–36]. Several miRNAs have been studied and confirmed as having a role in inflammatory processes in peripheral tissues such as adipose tissue and muscle [37]. Moreover, there is strong evidence that miRNAs would function as an effective system that regulates the magnitude of inflammatory responses, by displaying effects on cellular development and aspects of cellular function [38].

miRNAs-modulated pathways in skeletal muscle have been extensively studied. Several highly expressed miRNAs are described in striated muscle: the “myomiRs.” miRNAs such as miR-1, miR-133a, miR133-b, miR-206, miR-208, miR208b, miR486, and miR-499 are part of this group and are associated with cell growth and differentiation, stress responsiveness, and protection against apoptosis [39, 40]. Muscle protein degradation in cachexia is mainly mediated by the ubiquitin proteasome system, which is induced through the activation of E3 ligases, atrogin-1/MAFbx, and MurF-1. The Forkhead box O (FoxO) signaling pathway participates in this process by the induction of the transcription of E3 ubiquitin ligases and has three members in skeletal muscle (FoxO1, FoxO3, and FoxO4). Muscle-specific overexpression of these proteins is described as sufficient to cause skeletal muscle atrophy in vivo; and inhibition of FoxO transcription activity prevents muscle fiber atrophy during cachexia [41]. Xu and colleagues verified that the miRNA-486 decreases FoxO1 protein expression and promotes FoxO1 phosphorylation to suppress E3 ubiquitin ligases [42], presenting an excellent candidate for future studies on the mechanisms of regulation of muscle atrophy by miRNAs in cachexia. miR-206 and miR-21 were also recently described as having a role in muscle wasting in catabolic conditions [43]. miR-21 has been already confirmed as being produced and exported from tumor cells of rodent and humans and uptaken by the skeletal muscle, in exosomes. The effect of this process is the onset of proteolysis through toll-like receptor 7 signaling, in a JNK dependent manner [44]. Moreover, the detection of aberrant miRNA expression in body fluids, that is, blood, urine, and saliva, opens ways to explore these molecules as diagnostic and prognostic tools for cancer cachexia. miRNAs are also known to play a major role in the regulation of the transcription of several genes involved with key aspects of white adipose tissue metabolism. To date, one study involving cachectic patients, white adipose tissue, and miRNA profile is available in the literature [45]. In this study, five miRNAs showed specific cachexia associated patterns of expression. miR-483-5p/-23a/-744/-99b expression is downregulated and miR-378 is upregulated in cachectic patients. miR-378 is strongly involved with catecholamine-stimulated lipolysis in adipocytes and modulates the expression of key lipolytic proteins such as LIPE, PLIN1, and PNPLa2. No information is available in the literature about miRNAs expression and the modulation of inflammation in the white adipose tissue in specific wasting conditions. However, Xie et al., 2009 [46], demonstrated that a chronic inflammatory environment characterized by high cytokine concentration may, per se, change miRNA pattern expression in the white adipose tissue, both in cultured differentiated adipocytes and in rodent models. Potential miRNA candidates for studies regarding adipose tissue inflammation and cachexia would be miR-155, miR-146a, miR-21, and miR-9, whose expression is induced by the activation of innate immune system through toll-like receptors [47].

## 6. Conclusion

**Figure 3:**
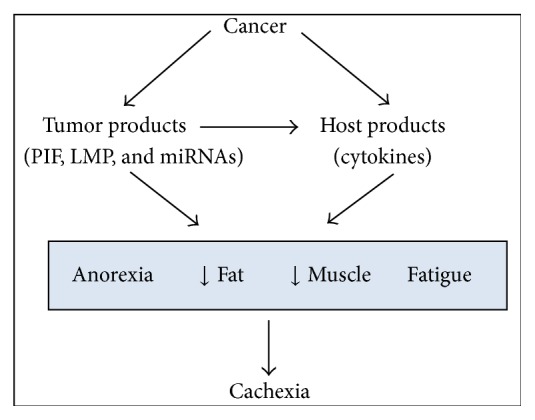
miRNA as part of cachexia modulation as an addition to the Tisdale proposition [8].

Considering that miRNAs are known to regulate the expression of genes involved in several types of diseases as cancer and autoimmune disorders [34, 35] and play a pivotal role in the regulation of inflammatory responses, the study of miRNAs in cachexia is a promising field of research, and patients could benefit not only from the development of new targets for treatments, but also from earlier diagnosis. Chronic inflammation in cancer cachexia is a highly complex biological process. The discovery of noncoding RNAs and the improvement of molecular biology techniques have changed the concept that inflammation could be understood and explained by the study of signaling pathways and by the contribution of specific proteins. Knowledge on the regulation of gene and protein expression has changed profoundly, and miRNAs are nowadays established as pivotal components of the signaling networks that modulate inflammatory processes [48], leading to wasting conditions such as cachexia. Based on such evidence, we propose that miRNAs participation in the onset and maintenance of cachexia should be added to the study of the syndrome (Figure 3).
